# Ambulance Crash Characteristics in the US Defined by the Popular Press: A Retrospective Analysis

**DOI:** 10.1155/2010/525979

**Published:** 2010-12-21

**Authors:** Teri L. Sanddal, Nels D. Sanddal, Nicolas Ward, Laura Stanley

**Affiliations:** ^1^Critical Illness & Trauma Foundation, 2135 Charlotte Street, Suite 2, Bozeman, MT 59718, USA; ^2^Western Transportation Institute, P.O. Box 174250, Bozeman, MT 59717, USA

## Abstract

Ambulance crashes are a significant risk to prehospital care providers, the patients they are carrying, persons in other vehicles, and pedestrians. No uniform national transportation or medical database captures all ambulance crashes in the United States. A website captures many significant ambulance crashes by collecting reports in the popular media (the website is mentioned in the introduction). This report summaries findings from ambulance crashes for the time period of May 1, 2007 to April 30, 2009. Of the 466 crashes examined, 358 resulted in injuries to prehospital personnel, other vehicle occupants, patients being transported in the ambulance, or pedestrians. A total of 982 persons were injured as a result of ambulance crashes during the time period. Prehospital personnel were the most likely to be injured. Provider safety can and should be improved by ambulance vehicle redesign and the development of improved occupant safety restraints. Seventy-nine (79) crashes resulted in fatalities to some member of the same groups listed above. A total of 99 persons were killed in ambulance crashes during the time period. Persons in other vehicles involved in collisions with ambulances were the most likely to die as a result of crashes. In the urban environment, intersections are a particularly dangerous place for ambulances.

## 1. Introduction

Ambulance crashes are too common in our national transportation system, especially in rural areas. The total number of ambulance crashes including minor “fender benders” per year has been estimated at 6,500 [1]. Vehicle performance standards, improper maintenance, variable operator training, and improper safety restraint use have been noted as contributing factors [2]. The occupational fatality rate from ambulance crashes is four times the US average when compared to other occupations [3]. Emergency medical personnel are at a higher crash risk than other first responders including law enforcement officers and firefighters [2]. The volunteer nature of the workforce [4–6], inadequate screening of vehicle operators [7, 8], inadequate vehicle operator training [7–14] fatigue and distraction [1, 7, 11, 15], poor knowledge of driving laws [16], poor vehicle design [7, 11, 17, 18], and inadequate policies and procedures have been linked to the increased crash rates. Unfortunately, little is known about ambulance crashes in general and rural ambulance crashes specifically [1, 2]. 

A non-peer-reviewed website, titled EMSNetwork (http://www.emsnetwork.org/), was identified as a primary source of information. EMSNetwork has been gathering articles from newspapers and other popular press sources concerning ambulance crashes since at least 2004. These newspaper and popular press articles were captured and analyzed using both qualitative and quantitative methods in an effort to further characterize ambulance crashes overall and to contrast differences that could be identified between urban and rural crashes. Website entries were examined retrospectively covering the period of May 1, 2007 to April 30, 2009. 

This paper represents the summation and analysis of 466 ambulance crash notices posted during that time period.

## 2. Methodology

The foundational literature search for this project was previously completed and resulted in a peer-reviewed publication [2]. During that review, it became clear that there were significant gaps in available data sets. The Fatal Analysis Reporting System of the National Highway Traffic Safety Administration captures a limited number of fatal ambulance crashes (approximately 27/year) [19]. However, it does not capture other major ambulance crashes including those that produce injuries to ambulance occupants or others. Some states maintain ambulance crash databases [20] but those rarely result in contributions to the peer-reviewed literature, nor are they readily known or available to researchers. The absence of a single database that captures all ambulance crashes nationally precludes our full understanding of the factors contributing to those crashes and the often fatal outcomes. A review of the extant literature confirms the need to gather descriptive data from all sources including the popular press. 

All ambulance crashes posted on the EMSNetwork website occurring between May 1, 2007 and April 30, 2009 were printed and abstracted by a single reviewer. According to the Editor-in-Chief of the EMSNetwork, its main methods of information gathering include, but are not limited to, “search engines, EMS groups/individuals, state EMS personnel, and the tenacious persistence of the EMSNetwork editors” [21].

A database was created in SPSS (v.16) to capture and analyze the data. The inclusion fields are described in Table 1. 

With the exception of urban/rural assignment, only data available from the press reports were used to populate the database for each incident. Urbanicity/rurality was assigned in a two-step process. First, the city or community location and state were entered into Google; from there the county of the incident was derived. The county was compared to a list of counties deemed eligible for funding from the Health Resources and Services Administration's Office of Rural Health Policy (available at http://ruralhealth.hrsa.gov/). If the county appeared on that list, it was indicated as a rural crash; if the county was not on the list the event was marked urban. 

Each printed report was abstracted by a single researcher (TLS). Data entry was accomplished by two individuals (CU and TLS) with data integrity checked by a single researcher (TLS). Due to the nature of the data source, every field could not be entered on each crash either because it did not pertain to that particular crash or it was not available in the media account. 

Analysis was largely descriptive in nature. Where analytic comparisons could be made significance was established at *P* = .05.

## 3. Results

From the dates May 2007 to April 2009, there were 466 ambulances crashes reported to this database. Of these, 358 (76.8%) resulted in injuries to persons inside or outside of the ambulance. Seventy-nine (79) crashes resulted in fatalities to persons inside or outside of the ambulance. Persons inside the ambulance included prehospital personnel, patients, or family members of patients being transported. Persons outside of the ambulance included those in other vehicles, pedestrians, and bystanders. A total of 99 deaths resulted from these fatal crashes. 

The number of reported crashes varied from 7 in June 2007, to 31 in January 2009. From May 2007 to April 2008, the monthly mean was 21, median was 22, and the dual mode was 17 and 24. The second year, from May, 2008 to April, 2009, monthly mean, median, and mode were 18, 18, and 14, respectively. The two year combined temporal distribution of crashes recorded mean was 19/month, median of 34/month, and mode 28/month. The two year combined temporal distribution of crashes by month showed slightly higher rates in the months of January (60), May (51), and December (47) when compared to the expected frequency. The months of March (28), April (28), and July (28) had slightly lower rates. 

**Figure 1:**
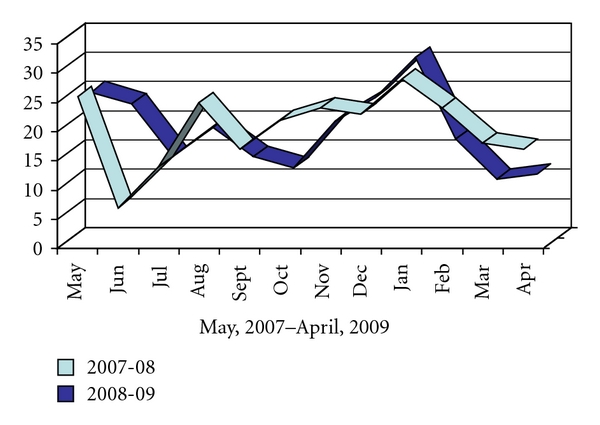
Distribution of ambulance crashes by month.

These temporal variations failed to reach statistical significance *χ* ^2^ (11, *N* = 12) = 14.6, *P* = .201. Likewise, there were no significant variation between the two years.  Figure 1 represents the frequency of ambulance crashes by month. 

**Figure 2:**
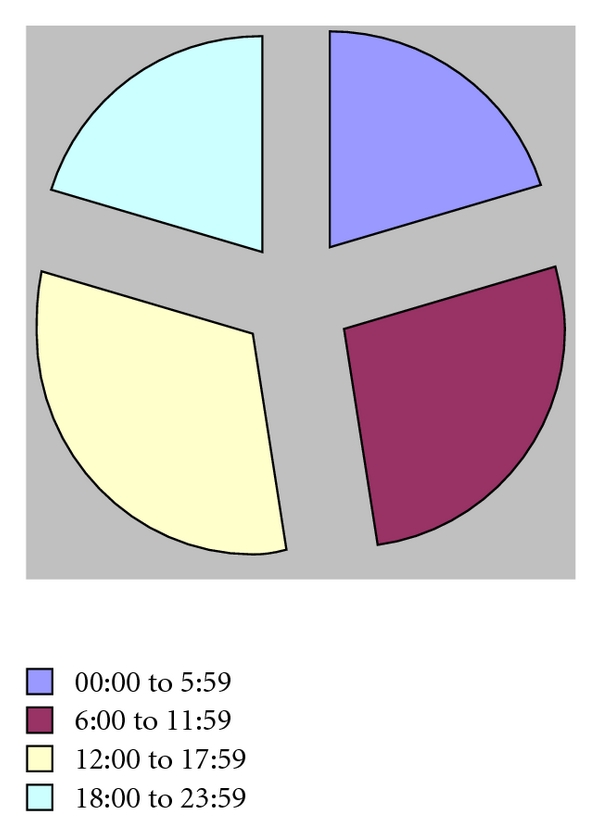
Time of crashes.

Time of the crashes was reported in 330 of the 466 crashes. Broad time periods were given in 10 cases, such as the crash occurred in the early morning hours. Distribution of crashes by a.m. to p.m. was similar with 157 crashes reportedly occurring in the morning hours (00:00–11:59) and 173 crashes occurred in the afternoon (12:00–23:59). Of those 320 cases where discrete times of the crash was noted, 67 crashes occurred between 00:00 to 5:59, 85 crashes between 06:00 and 11:59, 100 crashes occurred between 12:00 and 17:59, and 68 crashes happened between 18:00 and 23:59. As shown in Figure 2 below, time of crash appears equally distributed.

Road conditions were noted in 54 crash events. Of those, 51 (11% of 466 total) of the reports noted adverse conditions, including: 14 rain, 6 fog, 10 slippery, 1 whiteout/blizzard, 6 wet and icy, and 13 ice and snow. Where road conditions were noted, 3 of the events reportedly occurred on dry pavement. 

The nature of the ambulance operation at the time of the crash was noted in 214 accounts. Where noted, the ambulance was responding to an emergency in 145 (68%) of the events, was returning from a call in 25 (12%), and neither responding nor returning from a call in 44 (21%). This later category could involve the use of the ambulance for routine matters such as going to a meal or driving in a dynamic status management standby mode. In 111 (80%) of the 139 cases where it was noted, the ambulance had emergency warning devices (lights or lights and sirens) operating at the time of the crash. 

Whether or not the ambulance had a patient on board was captured in 340 (73% of 466 total) of the cases. In 178 (52%) of the cases no patient was on board at the time of the incident. The remainder 162 (48%) were transporting one or more patients at the time of the crash. 

Ambulances were reported as striking another vehicle or object in 150 (32%) crashes and being struck by another vehicle in 209 (45%) of crashes where such information was known. Many of these crashes involved more than one, but an unquantifiable, number of vehicles. 

Intersections were the most common location (196 (42%) of 466 total) noted for the crash. In 27 (14%) of these 196 intersection crashes, the ambulance rolled either onto its side or top. When filtered by intersection crash, in 69 (35%) of the 196, the ambulance was noted to be the striking vehicle. Rollover of the ambulance was noted as a feature of the crash in 49 additional nonintersection crashes. 

In 29 cases (6% of 466 total), the ambulance operator was found to be at fault and/or issued a citation for the crash. In 7 cases (2%), the ambulance operator was noted to have been over the legal limit for alcohol use. In 39 (8%) of the crashes, the driver of another vehicle involved in the crash was reported as cited for DUI. 

The distribution of crashes occurring in an urban or rural environment was noted to be 382 (82%) and 84 (18%) respectively. This proportion appears similar to the general population distribution of the US according to the urban/rural definitions selected for use in this study.

As a result of the 358 injury-producing crashes, a total of 982 persons were injured. The number of injuries per injury-producing crash varied from 1 to 13. The extent of the injuries was not known although the reports most frequently noted that the injured party was taken to a hospital, most often by another ambulance. Prehospital personnel were the most frequently injured persons in the crash with a total of 480 personnel being injured. In 172 (63%) of the 271 crashes in which prehospital personnel were injured, two or more prehospital personnel were injured. Drivers in other vehicles were injured the second most frequently. The total number of “others” injured was 431. The number of “others” injured per event ranged from 1 to 10. In 70 cases, the patient who was being transported suffered injuries or additional injuries. Eleven (11) pedestrians were struck by an ambulance. 

As a result of the 79 fatal crashes, a total of 99 persons died. Persons travelling in vehicles other than the ambulance were the most likely to die as a result of the event with 64 (65%) deaths in this category. This was followed by patients being transported at 19 (19%) and prehospital personnel at 14 (14%). The remaining 2 (2%) deaths were bystanders. 

**Figure 3:**
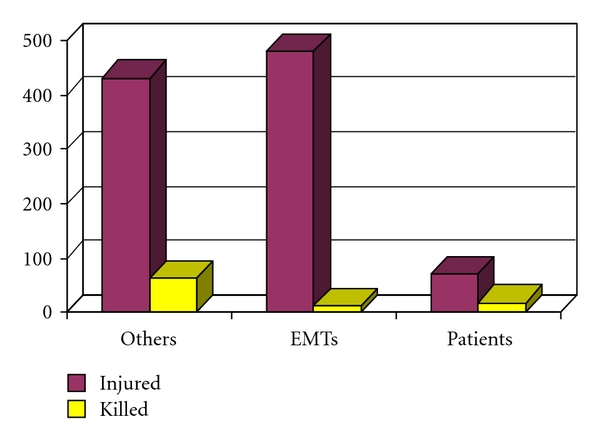
Comparison of injured and killed.

Figure 3 illustrates the distribution of persons injured with a comparison to categories of persons killed as a result of the 466 ambulance crashes. 

Only 5 crashes reported the ambulance having any form of quality feedback system, such as “black boxes” or video cameras. 

Information was known regarding the use of warning lights, or warning lights and sirens, in 112 cases. Of those 112 incidents, warning devices were being used at the time of the crash in 86 cases. One hundred percent of these (86) resulted in injuries and 23 resulted in fatalities. 

Table 2 summarizes the differences and similarities between urban and rural crash characteristics. Differences in the proportion of intersection crashes and non-intersection rollover crashes reached statistical significance with the rural environment having significantly fewer rollovers occurring at intersections.

## 4. Discussion

It appears that the database captures most, if not all, fatal ambulance crashes in the United States. Previous work by CDC [19] suggested that during the time period of 1991–2002 (11 years) the average number of fatal ambulance crashes reported to the US Department of Transportation's Fatality Analysis Reporting System (FARS) database was 27 per year. This compares favorably with the 46 and 33 fatal crashes per year identified through this data source. One of the limitations noted in the FARS analysis was that it was not always possible to identify whether the fatality involved a prehospital care provider. These data allowed for a more precise characterization of decedents as prehospital professionals, patients or family members riding in the ambulance, civilian personnel in other vehicles, or pedestrians. The analysis also allowed, for the first time in the literature, to describe the distribution on nonfatal injuries to prehospital personnel, other vehicle operations, patients being transported, and pedestrians. This information, to the best of our knowledge, is not available in any other data set of a national scope. 

The distribution of urban and rural crashes also tends to support the validity of the database. Nationally, it is estimated that 20% of the population live in rural areas and 80% are urbanites. These estimates correlate well with the distribution rates of 18% and 82% respectively for ambulance crashes contained in the database. 

**Figure 4:**
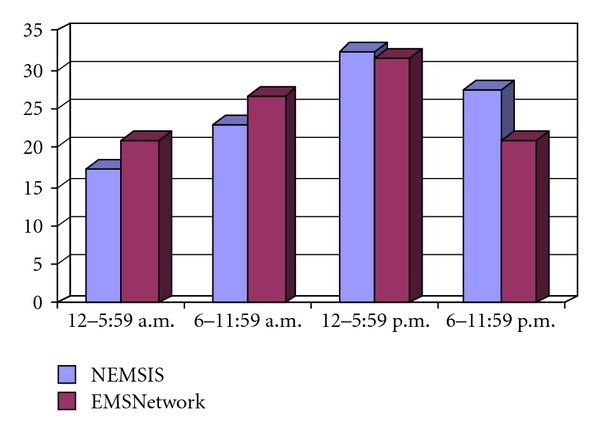
Comparison NEMSIS and EMSnetwork.

Additional confirmation of the validity of the database can be found in the similarities between the quartile temporal summaries of these data and their comparison with the National EMS Information System (NEMSIS) which shows a very similar distribution of the number of 9-1-1 EMS responses overall [22] (Figure 4). 

Certainly, the total number of crash records in the database annually, 246 and 220 respectively, do not come close to the 6500 ambulance crashes/year estimate suggested by Zagaroli and Taylor [1]. This could be reflective of either a gross overestimation by Zagaroli or, more likely, by the fact that most popular media, particularly those in larger urban markets, do not report on minor “fender benders” even if they do involve ambulances. 

The distribution of nonfatal injuries was an unexpected finding. Prehospital personnel operating the ambulance seem to be at greater risk of injury, in sheer numbers, than patients, persons in other vehicles, or pedestrians. This could relate to the fact that, while working in the rear compartment, many, if not most, prehospital personnel are not secured with occupant restraint systems [7–9, 13, 17, 19, 23–28]. It was also interesting that, in most injury producing crashes in which prehospital personnel are injured, 2 or more prehospital personnel are injured. Given the high numbers of prehospital personnel injured, the reason that they are the least likely group to suffer a fatality, surpassed by other vehicle occupants, patients, and pedestrians, is unclear. 

We expected to find differences between rural and urban ambulance crashes. With the exception of the geographic location of the crash in urban environments being intersections more frequently than in rural environments along with the preponderance of non-intersection rollovers in the rural environment, there were far more similarities than differences. The finding of non-intersection rollovers in rural environments may indicate an opportunity for focused driver's training on issues of off-road recovery maneuvers. It has long been the assertion in the literature that rural crashes more often involve rollovers and fatalities [23, 25, 29]. These data do not support those assumptions. While there is a difference in rollover type, as noted above, the proportion of rollovers in the two environments is similar but with distinctly different causes. In the urban environment, rollovers are most often due to the impact of another vehicle striking the ambulance. In rural environments, the rollovers do not involve intersection collisions. 

The other essential issue that is verified in the analysis of these data is the fact that the use of lights or lights and sirens often places the responding ambulance and the civilian population at risk. Prehospital professionals may make assumptions that the use of these warnings give them license to disregard certain rules of the road pertaining to intersection controls (stop signs and traffic signals) and direction of travel (against traffic). The civilian population is, clearly, underinformed on how to respond to visual and/or audible signals from emergency vehicles. Education of both populations is essential. Of particular note are the findings of several studies that the time saved by using lights or lights and sirens is insignificant to the outcome of the patient in nearly all cases. The recommendations of Sanddal et al. [2], that all jurisdictions should adopt and enforce policies concerning the use of lights and sirens, should be promoted. 

Driving under the influence (DUI) of drugs or alcohol, both in the civilian and prehospital provider populations, contributes to both fatal and injury-producing events. Thirty-nine civilians and seven EMTs were reported as DUI at the time of the crash. Persistent DUI enforcement programs among the civilian population and zero tolerance policies for prehospital personnel need to be continued and expanded. 

In only 5 of the fatal and the injury-producing crashes was it noted that quality feedback systems (QFS), (video cameras or “black box” type instruments) were in use in the ambulance. Even though this number may be skewed by the fact that ambulances equipped with such devices are less likely to be involved in a crash, Levick and Swanson [3] document the dramatic reduction in crashes following the implementation of QFS in an urban ambulance fleet. The continued deployment of QFS and the strict monitoring and enforcement of findings has a clear potential to substantially reduce serious ambulance crashes. 

### 4.1. Limitations

While the dataset used for this report represents the most complete collection of fatal and nonfatal ambulance crashes in the US, it is based on media reports which vary, to an unknown degree, in accuracy and completeness. The staff of the EMSNetwork site are responsible for “searching” for crashes or are reliant on the good will of the prehospital care community for the notification and documentation of events. A sampling bias could exist within the database. Communities where there is aggressive reporting by daily newspapers or a competitive television market may be overrepresented. Conversely, rural communities that have either no local paper or small weekly publications may be underrepresented. 

An additional sampling bias concern is made clear in that the database does not capture all crash events involving ambulance services. The discordance between the 466 cases in the database and the 13,000 expected cases over the two-year period according to Zagaroli's [1] estimates reflect the absence of many crash incidents. The distribution of those incidents by minor, injury-producing, and fatal cannot be estimated with any degree of certainty. 

The assignment of urban/rural occurrence was, at best, a gross reflection of those conditions. It is clear that within rural counties on the HRSA/ORHP list there are communities of up to 50,000 persons and, therefore, the street, intersection and traffic conditions may be more reflective of an urban event. Likewise, in many urban counties there are secondary and county roadways that may be more closely aligned with the rural environments. Additional specificity in rural/urban determination was not possible due to limitations of the data. 

Lastly, even tracking the data across a two year period only resulted in 79 fatal and 358 injury producing, non-fatal crashes. These numbers represent a relatively small sample size and findings can not be generated or suggested without the possibility of Type II error.

## 5. Conclusions

This study confirms the work of Ray and Kupas [12] and others that note that the most common geographic feature of ambulance crashes is intersections. It also supports those authors' contention that one of the most effective methods of reducing such crashes is the establishment and enforcement of a complete stop rule at intersections and traffic signals when requesting the right of way. 

In the interest of public, provider, and patient safety, all states should require mandatory and standardized reporting of any crash involving an ambulance. The National Highway Traffic Safety Administration, with the assistance of the National Association of State EMS Officials and other key stakeholders, should develop standardized electronic reporting definitions and transaction language to capture and record ambulance crashes.

Emergency Medical Dispatch training, including the use of prearrival instructions, should be promoted to encourage the nonuse of warning devices (lights and sirens) during responses to non-life-threatening “emergency scenes”. All EMS agencies should adopt and enforce emergency response policies that include the use of warning devices. 

And finally, there should be continued research regarding ambulance operations and implementation of effective solutions to reduce crashes and their severity.

## Figures and Tables

**Table 1:** Inclusion fields.

Definition	Data Type
Date crash occurred	MM/YY
State where crash occurred	String
Ambulance rolled	Dichotomous
Crash occurred at an intersection	Dichotomous
Number of others injured as a result of crashes	Numeric value
Number of EMT's injured	Numeric value
Patient in ambulance received additional injuries	Dichotomous
Injuries occurred by anyone as a result of the crash	Dichotomous
Total number of injuries per event	Numeric value
Ambulance struck other vehicle or object	Dichotomous
Ambulance struck by other vehicle	Dichotomous
Ambulance transporting a patient at the time of the crash	Dichotomous
ETOH present in other driver	Dichotomous
ETOH present in ambulance operator	Dichotomous
Ambulance responding to a call	Dichotomous
Ambulance using lights and sirens	Dichotomous
Ambulance returning from a call	Dichotomous
Ambulance not on duty	Dichotomous
Crash occurred in the A.M.	Time
Crash occurred in the P.M.	Time
Urban or rural setting (rural defined by HRSA/office of rural health policy)	Dichotomous
Road conditions	Pick List
Ambulance struck pedestrian	Dichotomous
Ambulance operator found at fault or issued citation	Dichotomous
Lawsuit instituted as a result of crash	Dichotomous
Death occurred by anyone as a result of the crash	Dichotomous
Total number of deaths per event	Numeric Value
Number of patients killed in crash	Numeric Value
Number of EMT's killed in crash	Numeric Value
Others killed in crash	Numeric Value

**Table 2:** Urban and rural crash characteristics.

Characteristic	Urban		Rural	
	Number	Percent	Number	Percent
Total crashes	382	82	84	18
Nonintersection rollover^†^	59	15	17	20
Intersection*	176	46	20	24
Injury crashes	298	78	60	71
Total injuries	824		148	
Fatal crashes	63	17	16	19
Total fatalities	76		23	
Patient on board	60	15	23	27
Pt. additional injury	57	15	12	14
Lights and sirens	95	25	17	20

^†^Significant at *P* = .037  *χ* ^2^  (1, *N* = 103) = 4.81.  *P* = .037.

*Significant at *P* = .007  *χ* ^2^  (1, *N* = 466) = 6.37.*P* = .007.
